# Supplementary material for: Epidermal growth factor receptor signaling governs the host inflammatory response to invasive aspergillosis
Source: mBio. 2024 Oct 30;15(12):e02671-24. doi: 10.1128/mbio.02671-24 (PMC11633379; doi:10.1128/mbio.02671-24)
Supplement: Supplemental figures — Figures S1-S6. [file mbio.02671-24-s0001.pdf]

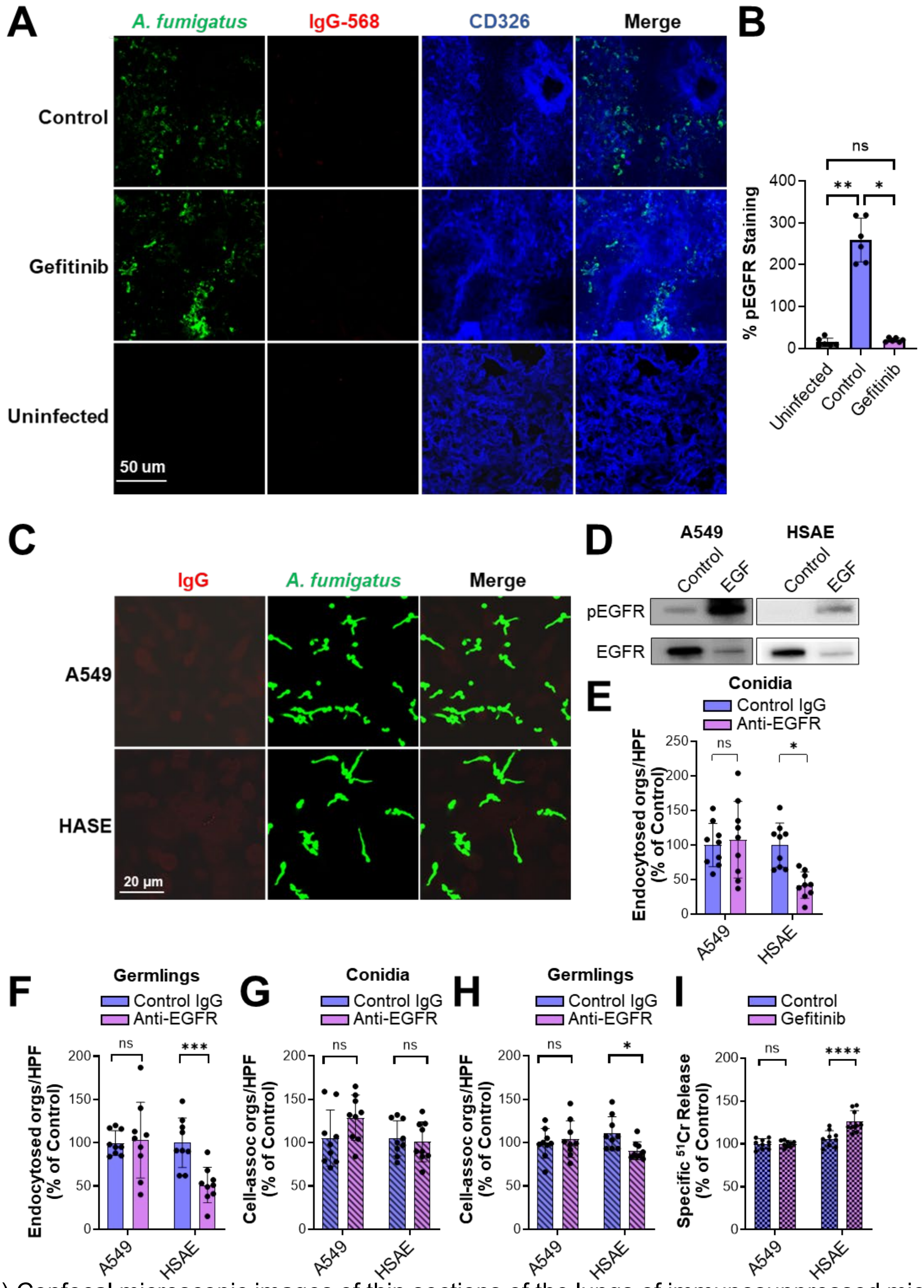

FIG S1 (**A**) Confocal microscopic images of thin sections of the lungs of immunosuppressed mice after 12 h of infection with *A. fumigatus.* The sections were stained for *A. fumigatus* (green), pulmonary epithelial cells (CD326; blue), and control rabbit IgG, (red). Scale bar: 50 µm.(**B**) Quantitative analysis of pEGFR immunofluorescence of images such as in Fig. 1C. Results are mean ± SD of 2 images per mouse from 3 mice per group. (**C**) Confocal micrographs showing A549 and HSAE cells infected with *A. fumigatus* for 2.5 h. Sections were stained with control rabbit IgG (red). Scale bar: 20 µm. (**D**) Western blots showing that exposure to epidermal growth factor (EGF) for 5 min stimulates EGFR phosphorylation in both A549 and HSAE cells. (**E** and **F**) Effects of an anti-EGFR antibody on the endocytosis of *A. fumigatus* conidia (**E**) and germlings (**F**) by A549 and HSAE cells. (**G** and **H**) Effects of an anti-EGFR antibody on the cell-association (a measure of adherence) of *A. fumigatus* conidia (**G**) and germlings (**F**) with A549 and HSAE cells. (**I**) Effects of the EGFR inhibitor gefitinib on *A. fumigatus*-induced damage to A549 and HSAE cells after 20 h. Results in (**E-I**) are mean ± SD of 3 independent experiments, each performed in triplicate. Orgs/HPF, organisms per high powered field; ns, not significant; *$P < 0.05$; ***$P < 0.001$; ****$P < 0.0001$ by one way ANOVA with Dunnett's test for multiple comparisons.

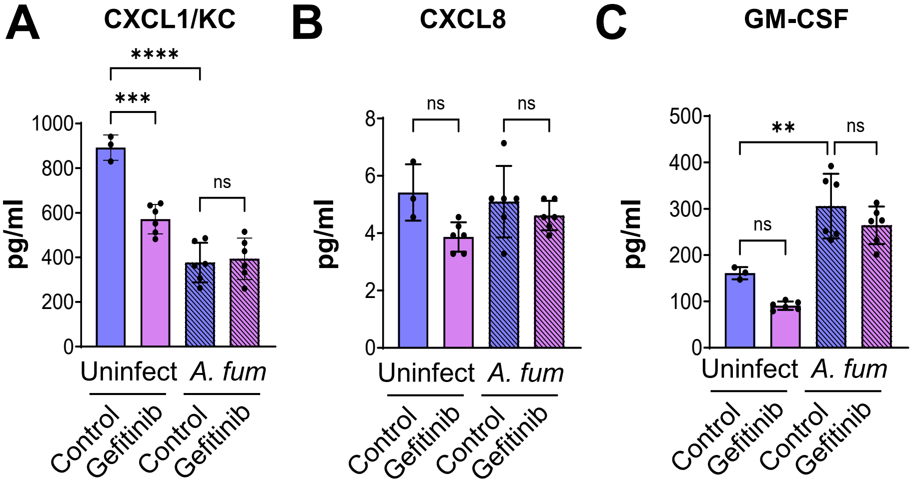

FIG S2 Effects of gefitinb **on** cytokine release in A549 cells infected with *A. fumigatus*. A549 cells were treated with gefitinib or DMSO control, infected with *A. fumigatus* for 16 h, and then levels of the indicated cytokines were measured. Results are mean ± SD of 3 independent experiments, each performed in duplicate. *A. fum, A. fumigatus;* uninfect, uninfected; ns, not significant; ***P* < 0.01; ****P* < 0.001; *****P* < 0.0001 by one way ANOVA with the Dunnett's test for multiple comparisons.

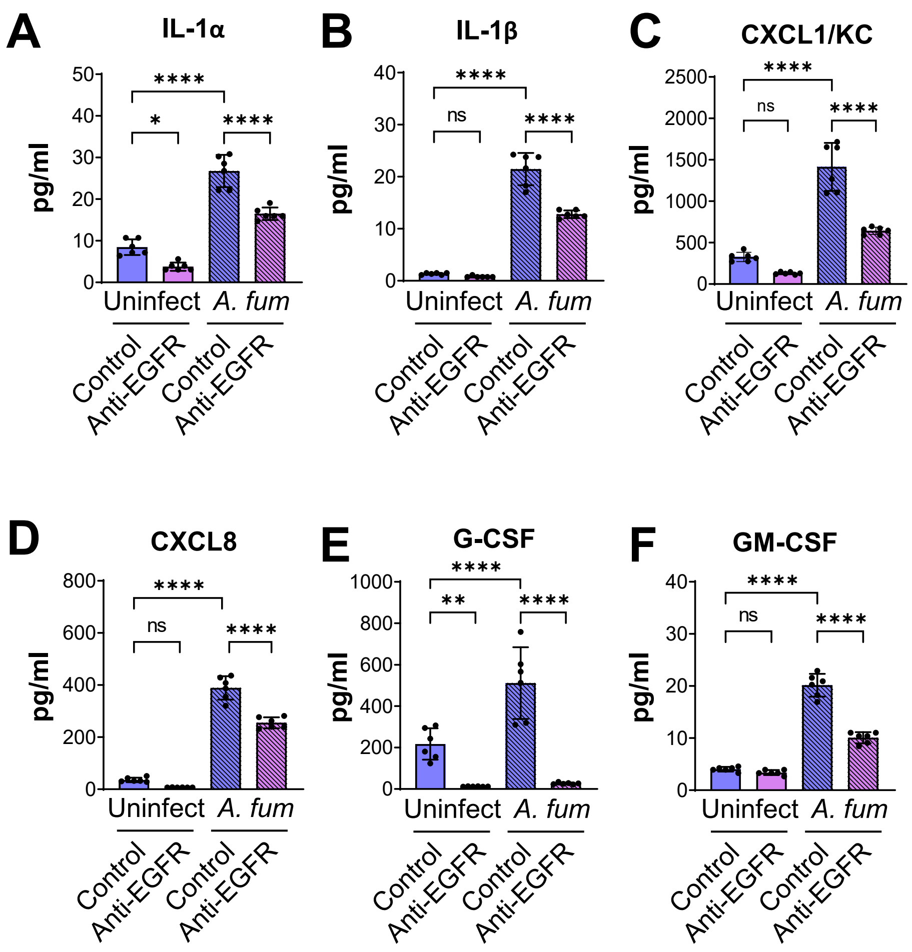

FIG S3 Effects of an anti-EGFR antibody on cytokine production by HSAE cells. HSAE cells were treated with an anti-EGFR antibody or control IgG, infected with *A. fumigatus* for 16 h, and then levels of the indicated cytokines were measured. Results are mean ± SD of 3 independent experiments, each performed in duplicate. *A. fum, A. fumigatus;* uninfect, uninfected; ns, not significant; $^{**}P < 0.01$; $^{****}P < 0.0001$ by one way ANOVA with the Dunnett's test for multiple comparisons.

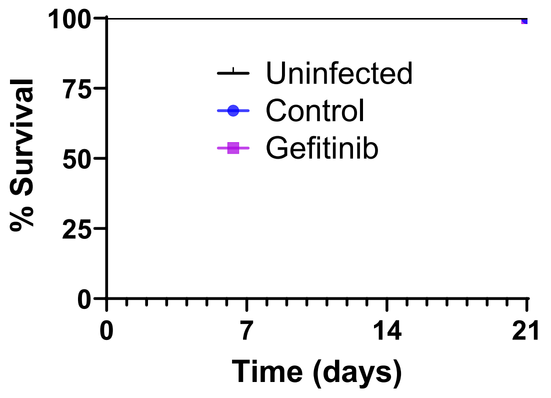

FIG S4 Effects of gefitinib on the survival of immunocompetent mice infected intratracheally with *A. fumigatus.* Results are from 8 mice per group in a single experiment.

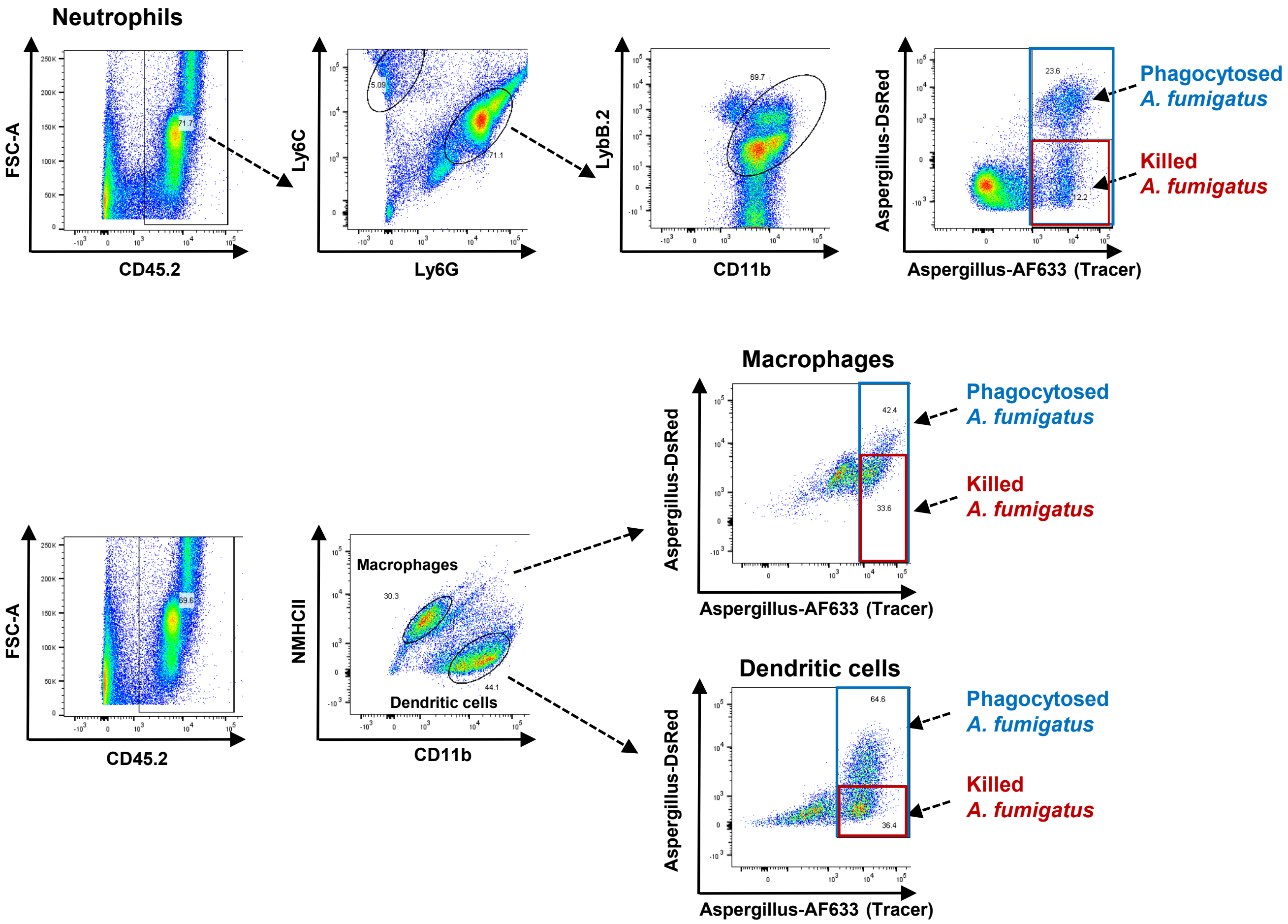

FIG S5 Gating strategy of flow cytometric analysis of *A. fumigatus* phagocytosis and killing by immune cells in the lung.

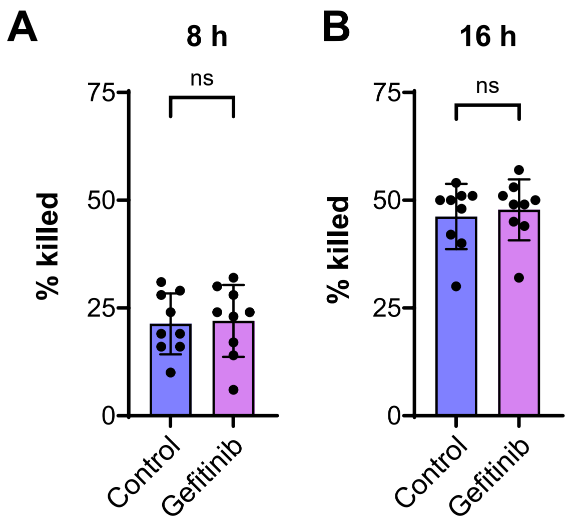

FIG S6 (**A** and **B**) Effects of gefitinib on *A. fumigatus* killing by bone marrow-derived macrophages after 8 (**A**) and 16 (**B**) h of incubation**.** Results are the mean ± SD of 3 experiments each performed in triplicate. ns, not significant by two-way Students t-test.
